# Rigidez Aórtica por Ressonância Magnética Cardíaca: Ferramenta Prognóstica ou Mero Espectador?

**DOI:** 10.36660/abc.20220231

**Published:** 2022-05-04

**Authors:** Sérgio Figueiredo Câmara, Henrique Barbosa Ribeiro

**Affiliations:** 1 Instituto do Coração Universidade de São Paulo São Paulo SP Brasil Instituto do Coração (InCor), Universidade de São Paulo, São Paulo, SP – Brasil; 2 Hospital Samaritano Paulista São Paulo SP Brasil Hospital Samaritano Paulista, São Paulo, SP – Brasil

**Keywords:** Análise de Onda de Pulso/métodos, Rigidez Aórtica, Diagnóstico por Imagem, Imagem por Ressonância Magnética/métodos, Prognóstico, Rigidez Vascular

A rigidez arterial aumenta com a idade e pode estar relacionada a taxas mais altas de eventos cardiovasculares, incluindo mortalidade.^1 - 4^ Essa capacidade preditiva foi demonstrada em várias coortes longitudinais, incluindo estudos populacionais de comunidades ‘saudáveis’ e pessoas com diabetes, hipertensão, doença renal crônica e doença arterial coronariana estabelecida.^5 , 6^ Existem várias maneiras de medir a rigidez arterial, como ultrassom-doppler, tonometria carótida-femoral e ressonância magnética cardíaca (RMC). A RMC fornece informações sobre a função cardíaca, perfusão e cicatrização miocárdica em um único exame e também pode ser o método preferido para avaliar a rigidez arterial usando a velocidade da onda de pulso aórtica (VOP).^7 - 9^ Embora tenha sido demonstrada a associação entre rigidez aórtica e isquemia miocárdica, bem como o valor prognóstico da rigidez aórtica pela RMC,^7^ há dados limitados sobre o valor prognóstico da VOP por RMC em pacientes idosos nos quais as doenças cardiovasculares (DCV) são responsáveis pela grande maioria das causas de mortalidade.

Nesta edição da revista, Kaolawanich e Boonyasirinant^10^ avaliaram a ocorrência de eventos cardíacos e cerebrovasculares adversos maiores (ECCAM), incluindo mortalidade cardíaca, infarto do miocárdio não fatal, hospitalização por insuficiência cardíaca, revascularização tardia (>180 dias após RMC) e acidente vascular cerebral isquêmico em pacientes idosos (> 70 anos) com suspeita ou DAC confirmada, submetidos a RMC de estresse com adenosina, incluindo a VOP. O objetivo principal foi determinar o valor prognóstico da rigidez aórtica usando VOP baseada em RMC em pacientes idosos com DAC. Duzentos e sessenta e três pacientes consecutivos (55% do sexo feminino; 77±5 anos) entre 2010 e 2014 foram incluídos com acompanhamento médio de 59,6 meses e VOP média de 13,98 ± 9,00 m/s. Uma VOP maior (>13,98 m/s) foi associada a maiores taxas de ECCAM (FC 1,75; IC 95% 1,05 - 2,94; p=0,03), em comparação com VOP não elevada (<13,98 m/s). Por análise multivariada, pressão arterial diastólica, fração de ejeção do ventrículo esquerdo (FEVE), isquemia miocárdica e VOP elevada foram preditores independentes de ECCAM no seguimento de longo prazo (p<0,05 para todos). A VOP teve valor prognóstico incremental em relação à história clínica, FEVE e isquemia (aumento do qui-quadrado global = 7,25; p=0,01). Nesta avaliação, pacientes idosos com VOP elevada também apresentaram maior prevalência de hipertensão, diabetes mellitus e pressão arterial sistólica mais elevada do que aqueles com VOP não elevada, consistente com estudos prévios em populações mais jovens.^11^

Alguns aspectos do trabalho de Kaolawanich e Boonyasirinant e a avaliação por RMC da VOP, merecem uma discussão mais aprofundada. Primeiro, a medida da VOP por RMC pode ser um dos métodos preferidos para avaliar a rigidez aórtica, pois oferece alta resolução, sem radiação ionizante,^10^ e ao contrário da VOP carotídeo-femoral usando tonometria, a RMC pode medir a distância aórtica sem suposições geométricas.^11^ Da mesma forma, consistente com estudos anteriores, a VOP medida pela RMC teve excelente reprodutibilidade.^3 , 11 , 12^ A VOP foi mensurada durante o período de avaliação da viabilidade e estresse, e a técnica de não apneia se mostrou conveniente para esses pacientes. Notadamente, as imagens de VOP foram adquiridas aproximadamente 10 minutos após a injeção de adenosina. No presente estudo, o valor médio de 13,98 m/s foi utilizado como ponto de corte para determinar pacientes com maior rigidez arterial. Estudos anteriores usaram vários valores de corte para VOP em pessoas mais velhas/idosas sem doença cardiovascular, variando de 9,5 a 13,2 m/s. No entanto, nenhum ponto de corte padronizado foi determinado para VOP usando RMC para as diferentes populações. Além disso, como este estudo foi realizado em idosos asiáticos, a possibilidade de generalização dos dados para pacientes mais jovens e de outra etnia também é incerta.

Outro aspecto importante do presente estudo é que a VOP mais alta resultou em taxas ~2 vezes maiores de ECCAM, com valor prognóstico incremental sobre variáveis clínicas e RMC, incluindo FEVE e isquemia miocárdica. Os principais fatores que aumentaram as taxas de ECCAM foram acidente vascular cerebral isquêmico (8,4% vs. 2,2%; p=0,01), consistente com dados anteriores.^2 , 13 , 14^ Ressaltam-se também as taxas de mortalidade semelhantes de acordo com as diferentes taxas de VOP. Vários estudos investigaram o valor prognóstico da rigidez arterial em diferentes populações com certas inconsistências. Enquanto estudos anteriores encontraram uma associação entre rigidez arterial e eventos cardiovasculares,^2 , 14 , 15^ essa associação apareceu limitada em outro estudo, especialmente para a população mais idosa.^11^ Portanto, o real impacto da rigidez arterial nas taxas de ECCAM em populações mais velhas, principalmente em relação à mortalidade (global e cardiovascular), merecerá confirmação adicional de estudos maiores.

Em conclusão, a rigidez aórtica pela RMC pode ser um marcador prognóstico adicional de eventos cardiovasculares em pacientes idosos com DAC suspeita ou confirmada. No entanto, estudos maiores com uma população mais heterogênea incluindo várias etnias devem confirmar tais achados e determinar o ponto de corte da VOP mais adequado relacionado a um pior prognóstico. O trabalho de Kaolawanich e Boonyasirinant certamente esclareceu sobre a importância da rigidez aórtica no arsenal das já vastas possibilidades diagnósticas e prognósticas da RMC em pacientes com suspeita de DAC. Resta saber se a rigidez aórtica será uma ferramenta prognóstica adicional ou um mero espectador na prática clínica, bem como se o manejo clínico desses pacientes com maior rigidez aórtica deva ser modificado.
