# Supplementary material for: Rigidez Aórtica por Ressonância Magnética Cardíaca: Ferramenta Prognóstica ou Mero Espectador?
Source: Arq Bras Cardiol. 2022 May 4;118(5):972–3. [Article in Portuguese] doi: 10.36660/abc.20220231 (PMC9368888; doi:10.36660/abc.20220231)
Supplement: Translation

# Translation: Aortic Stiffness by Cardiac Magnetic Resonance: Prognostic tool or Bystander?

**Authors:** Sérgio Figueiredo Câmara, Henrique Barbosa Ribeiro

**Affiliations:** 1 Brazil Instituto do Coração (InCor), Universidade de São Paulo, São Paulo, SP – Brazil; 2 Brazil Hospital Samaritano Paulista, São Paulo, SP – Brazil

**Keywords:** Pulse Wave Analysis/methods, Aortic Stiffness, Diagnostic Imaging, Magnetic Resonance Imaging/methods, Prognosis, Vascular Stiffness

Arterial stiffness increases with age and may relate to higher rates of cardiovascular events, including mortality.^1 - 4^ This predictive capacity has been demonstrated in various longitudinal cohorts, including ‘healthy’ community population studies and those with diabetes, hypertension, chronic kidney disease, and established coronary artery disease.^5 , 6^ There are several ways to measure arterial stiffness, such as doppler-ultrasound, carotid-femoral tonometer, and cardiac magnetic resonance (CMR). CMR provides information regarding cardiac function, perfusion, and myocardial scarring in a single exam and may also be the preferred method for assessing arterial stiffness using aortic pulse wave velocity (PWV).^7 - 9^ While the association between aortic stiffness and myocardial ischemia has been demonstrated, as well as the prognostic value of aortic stiffness using CMR,^7^ there is limited data regarding the prognostic value of PWV by CMR in elderly patients in whom cardiovascular diseases (CVD) account for the vast majority of mortality causes.

In this issue of the journal, Kaolawanich and Boonyasirinant^10^ evaluated the occurrence of major adverse cardiac and cerebrovascular events (MACCE), including cardiac mortality, nonfatal myocardial infarction, hospitalization for heart failure, late revascularization (>180 days after CMR) and ischemic stroke in elderly patients (> 70 years) with suspected or confirmed CAD undergoing adenosine stress CMR including PWV. The main objective was to determine the prognostic value of aortic stiffness using CMR-based PWV in elderly patients with CAD. Two hundred sixty-three consecutive patients (55% female; 77±5 years) between 2010 and 2014 were included with a median follow-up of 59.6 months and a mean PWV of 13.98 ± 9.00 m/s. A higher PWV (>13.98 m/s) was associated with greater MACCE rates (HR 1.75; 95% CI 1.05 - 2.94; p=0.03), as compared to non-elevated PWV (<13.98 m/s). By multivariable analysis, diastolic blood pressure, left ventricular ejection fraction (LVEF), myocardial ischemia and elevated PWV were independent predictors of MACCE at long-term follow-up (p<0.05 for all). PWV had an incremental prognostic value concerning clinical history, LVEF and ischemia (increased global chi-square = 7.25; p=0.01). In this evaluation, elderly patients with elevated PWV also had a higher prevalence of hypertension, diabetes mellitus and higher systolic blood pressure than those with non-elevated PWV, consistent with prior studies in younger populations.^11^

Some aspects of Kaolawanich and Boonyasirinant’s work and CMR evaluation of PWV merit further discussion. First, measurement of PWV using CMR might be one of the preferred methods for assessing aortic stiffness as it offers high resolution, without ionizing radiation,^10^ and unlike carotid-femoral PWV using tonometry, CMR can measure aortic distance without geometric assumptions.^11^ Likewise, consistent with previous studies, PWV measured by CMR had excellent reproducibility.^3 , 11 , 12^ PWV was measured during the period of viability and stress studies, and the non-breath holding technique proved to be convenient for such patients. Notably, PWV images were acquired approximately 10 minutes after adenosine injection. In the present study, the mean value of 13.98 m/s was used as the cut-off to determine patients with higher arterial stiffness. Prior studies have used various cut-off values for PWV in older/elderly adults without cardiovascular disease, ranging from 9.5-13.2 m/sec. Nevertheless, no standard cut-off level has been well determined for PWV using CMR for the different populations. Furthermore, as this study has been conducted among elderly Asian patients, the possibility of generalizing the data to younger patients and those from another ethnicity is also uncertain.

Another important aspect of the present study is that higher PWV resulted in ~2-fold higher rates of MACCE, with an incremental prognostic value over clinical and CMR variables, including LVEF and myocardial ischemia. The main factors increasing MACCE rates were ischemic stroke (8.4% vs. 2.2%; p=0.01), consistent with previous data.^2 , 13 , 14^ It should also be underlined the similar mortality rates according to the different PWV rates. Several studies have investigated the prognostic value of arterial stiffness in different populations with certain inconsistencies. While prior studies found an association between arterial stiffness and cardiovascular events,^2 , 14 , 15^ this association appeared limited in another study, especially for the older population.^11^ Therefore, the real impact of arterial stiffness on MACCE rates in older populations, especially regarding mortality (global and cardiovascular), will merit further confirmation from larger studies.

In conclusion, aortic stiffness using CMR could be an additional prognostic marker of cardiovascular events in elderly patients with suspected or confirmed CAD. However, larger studies with a more heterogeneous population with various ethnicities should confirm such finding and further determine the more appropriate cut-off point of PWV related to a worse prognosis. The work by Kaolawanich and Boonyasirinant has certainly shed some light on the importance of aortic stiffness in the armamentarium of the already vast diagnostic and prognostic possibilities of CMR among patients with suspected CAD. Whether aortic stiffness will be an additional prognostic tool or a mere bystander in clinical practice remains to be determined, as well as by what matters the clinical management of such patients with a higher aortic stiffness should be further modified.
